# The Role of miRNA-146a and Proinflammatory Cytokines in Carotid Atherosclerosis

**DOI:** 10.1155/2020/6657734

**Published:** 2020-12-09

**Authors:** Pan Huang, Xiao-ying He, Min Xu

**Affiliations:** ^1^Department of Neurology, People's Hospital of Deyang City, 173 Taishan North Road, Deyang, Sichuan 618000, China; ^2^Department of Neurology, The Affiliated Hospital of Southwest Medical University, No. 25, Taiping Street, Jiangyang District, Luzhou, Sichuan Province, Sichuan 646000, China; ^3^Department of Neurology, The Second People's Hospital of Deyang City, 340 Minjiang West Road, Deyang, Sichuan 618000, China

## Abstract

The aim of this study was to investigate the expression and significance of miRNA-146a in peripheral blood of patients with carotid atherosclerosis (CAS). Patients with CAS were selected as the stenosis (CAS) group (*n* = 180). According to the degree of stenosis, they were divided into the mild stenosis group (MS group, *n* = 64), moderate stenosis group (M group, *n* = 62 cases), and severe stenosis group (SS group, *n* = 54). According to the plaque type, patients were divided into a stable plaque group (SP group, *n* = 96) and a vulnerable plaque group (VP group, *n* = 84). Patients without CAS represented the normal group (*n* = 90). The expression levels of miRNA-146a as well as IL-6 and TNF-*α* in peripheral blood were detected by RT-PCR and ELISA, respectively. The expression levels of miRNA-146a, IL-6, and TNF-*α* in the CAS group were higher than those in the normal group and positively correlated with the degree of stenosis and plaque vulnerability. The expression levels of miRNA-146a, IL-6, and TNF-*α* in the stable plaque group were lower than those in the vulnerable plaque group. The area under the curve of miRNA-146a predicting the vulnerability of plaques was significant at 0.641. The expression level of miRNA-146a in the CAS group was positively correlated with IL-6 and TNF-*α* levels. Our results indicate that miRNA-146a may participate in the occurrence and development of CAS by regulating the expression of IL-6 and TNF-*α*, which may be potential biomarkers of CAS.

## 1. Introduction

Carotid atherosclerosis (CAS) is the leading cause of cerebral infarction. Studies have shown that the rate of carotid stenosis is 50−69%, 70−89%, and 90% of the following annual risks of stroke: 0.8%, 1.4%, and 1.9%, respectively [1]. The rupturing of vulnerable plaques in cerebral blood vessels is the main mechanism of cerebral infarction [2] [3]. Therefore, early assessment of the degree of stenosis, CAS, and plaque vulnerability is important for preventing and reducing cerebral infarction. DSA is currently the “gold standard” with high sensitivity for CAS diagnosis; however, its general development is more difficult, and carotid ultrasound and MRI are not good for predicting early CAS or plaques. Due to the shortcomings of imaging technology, researchers have turned their attention to circulating blood biomarkers. CAS has been widely recognized as an inflammatory reactive disease [4], and active inflammatory responses also increase plaque vulnerability [5], suggesting that inflammatory cytokines may be potential biomarkers for predicting CAS. However, subsequent studies demonstrated that the sensitivity and specificity of inflammatory cytokines to CAS are not high [6].

With the development of genetic technology, microRNA (miRNA) has gradually gained attention [7]. miRNA can be stably expressed in peripheral blood and has become a biological marker of various diseases. miRNA-146a has been shown to regulate inflammatory responses, which are associated with the process of CAS [8]. Therefore, we postulated that there is a differential expression profile of miRNA-146a in CAS patients. Furthermore, we investigated whether miRNA-146a expression is related to the degree of CAS stenosis and plaque vulnerability and whether miRNA-146a participates in the regulation of inflammatory responses and thus affects the occurrence of CAS and plaque vulnerability. To date, no study has addressed these mechanisms, and therefore, we investigated the expression of miRNA-146a in peripheral blood of CAS patients and its relationship with CAS stenosis, plaque vulnerability, and inflammatory cytokines. These findings may facilitate the discovery of biomarkers that could predict the degree of CAS stenosis and plaque vulnerability in the early stages of the disease.

## 2. Materials and Methods

### 2.1. Study Type

This study is a Nonrandomized Controlled Trial (nRCT).

### 2.2. Research Subjects and Clinical Data

**Figure 1:**
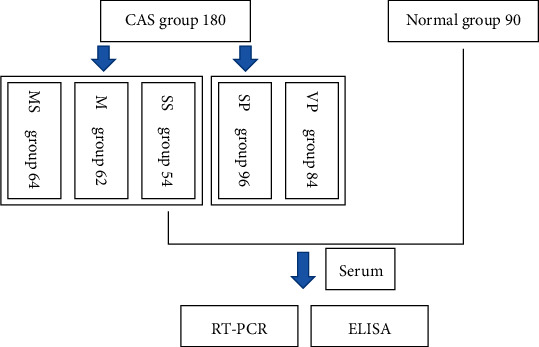
Flow chart of the inclusion process. MS: mild stenosis; M: moderate stenosis; SS: severe stenosis; SP: stable plaque; VP: vulnerable plaque.

In this study, 180 patients with CAS who were diagnosed by using carotid artery color Doppler in Deyang City People's Hospital were selected for the stenosis group. The inclusion criteria were as follows: there is compliance with color Doppler diagnosis for carotid intima-media thickening and atherosclerotic plaque formation, CT or MRI examination showed no cerebral infarction, cerebral hemorrhage, or other diseases, at least one risk factor was present (e.g., high blood pressure, diabetes, smoking, obesity, and hyperlipidemia), and patients are conscious and could cooperate until the test completion. The exclusion criteria were as follows: recent signs and symptoms of infection after relevant examinations, heart, liver, or kidney dysfunction, a history of combined immune system diseases and familial genetic diseases, malignant tumors or mobility problems, antibiotic use, and communication disorders that could prevent cooperation in the trial. According to the symptomatic classification of carotid stenosis in North America, the patients were divided into the following stenosis groups: mild stenosis group (MS group, *n* = 64), moderate stenosis group (M group, *n* = 62), and severe stenosis group (SS group, *n* = 54). At the same time, all carotid ultrasounds were divided according to plaque characteristics, resulting in a stable plaque group (SP group, *n* = 96) and a vulnerable plaque group (VP group, *n* = 84). Patients without CAS (*n* = 90) were selected for the control group (Figure 1).

In order to avoid bias, there was no statistically significant difference in age, gender, or risk factors between the stenosis and control groups (*P* > 0.05).

### 2.3. Specimen Collection and Serum Separation

In the morning (after fasting), 5 ml samples of peripheral venous blood of the patient was taken and allowed to naturally coagulate for 10−20 min at room temperature. The coagulated blood sample was centrifuged for 30 min at 3000 rpm. One part of the supernatant was immediately used to detect routine clinical biochemical indicators (Beckman LX20 fully automated biochemical analyzer, USA), and the other part (peripheral blood mononuclear cell (PBMC)) was stored at -80°C for total RNA extraction, RT-PCR, and ELISA.

### 2.4. RT-PCR Detection of miRNA-146a Relative Expression

After extracting total RNA according to the TRIzol kit (Invitrogen™, 15596-026), the value of O_260_/O_280_ measured by the nucleic acid protein analyzer (Shanghai Qi Te, WXJ-9388) was regarded as quality RNA at 1.8–2.0, and then, M was used. The MLV reverse transcriptase kit for cDNA synthesis was used (Invitrogen™, C-28025). After first-strand cDNA synthesis, the RT-PCR reaction mixture was placed in 96 wells of a PCR machine (Hangzhou Bori Technology, TC-XP). The following were mixed in the reaction plate: 5 *μ*l 2x PCR Mix, 1 *μ*l primer working solution (2.5 *μ*M), 1 *μ*l template, 2.8 *μ*l ddH_2_O, 0.2 *μ*l Rox, and 10 *μ*L total volume, with three replicate wells per sample. The amplification cycle conditions were 95°C (1 min), 95°C (15 s), 58°C (20 s), and 72°C (45 s), for a total of 40 cycles. The miRNA-146a primer sequence was as follows: forward: 5′-CCTGAGAAGTGAATTCCATGGG-3′ and reverse: 5′-CTCAACTGGTGTCGTGGAGTC-3′; the internal reference gene U6 primer sequence was as follows: forward: 5′-CTCGCTTCGGCAGCACAT-3′ and reverse: 5′-AACGCTTCACGAATTTGCGT-3′. Since the Ct value in RT-PCR cannot be used as the original data for statistical analysis, 2^-*△*Ct^ was used to represent the relative expression level of miRNA. ΔCt = (miRNA − 146a Ct value − U6 Ct value).

### 2.5. miRNA-146a Stability Test

**Figure 2:**
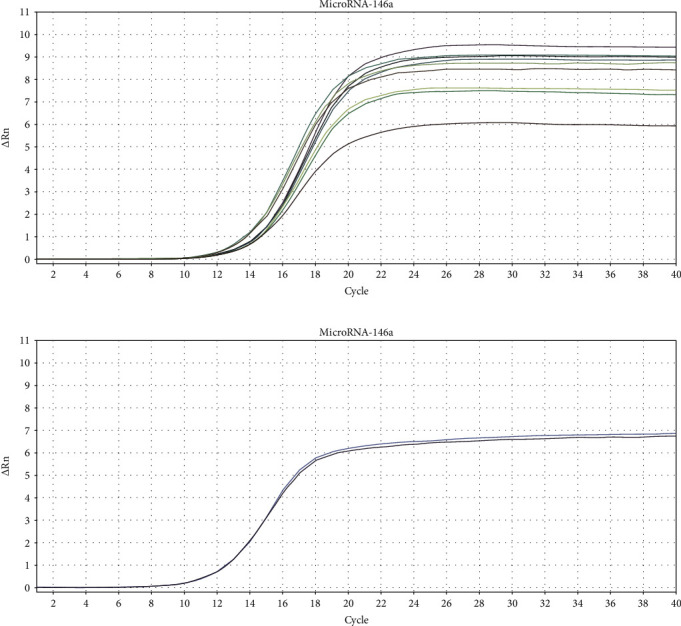
(a) MicroRNA-146a amplification curve in the CAS group. (b) MicroRNA-146a amplification curve in the normal group.

In order to stably detect miRNA-146a in circulating blood, we performed RT-PCR pretests on three patients with CAS and three patients from the normal group. It was found that RT-PCR started to expand around the 14^th^ cycle. After 28 cycles of amplification, the Ct value was meaningful, indicating that the selected five miRNAs met the requirements and the experimental conditions were well controlled (Figures 2(a) and 2(b)).

### 2.6. ELISA Detection of IL-6 and TNF-*α*

ELISA was used to detect the expression level of IL-6 and TND-*α* in peripheral blood of the CAS group and normal group (Wuhan Baiyier Biotechnology Co., Ltd., China).

### 2.7. Statistical Analysis

All data in this study were statistically analyzed with the SPSS 17.0 software. The measured data were expressed as mean ± standard deviation (*x* ± s). The paired *t*-test was used to compare two groups. The comparison between groups was performed by the independent sample *t*-test. For one-way ANOVA, the variance was tested using the rank sum test. The count data was expressed as the composition ratio or rate (%), and the chi-square (*x*^2^) test was used to compare the count data. Data were compared using the Wilcoxon two-sample rank sum test comparison method. ROC curves were drawn to evaluate the value of miRNA prediction of CAS plaque vulnerability. Correlation analysis was performed using the Pearson analysis. *P* < 0.05 was considered statistically significant.

## 3. Results

### 3.1. Comparison of Baseline Data

Comparison of baseline data showed that there was no significant difference in age, gender, or smoking between the CAS and normal groups (*P* > 0.05).

### 3.2. Analysis of CAS Risk Factors

Multivariate logistic regression analysis was performed using the factors in the baseline data as independent variables and carotid stenosis as a dependent variable. It was found that hypertension and high LDL-C levels were risk factors for CAS, while high HDL-C levels were protective factors for CAS (*P* < 0.05) (Table 1).

### 3.3. Comparison of miRNA-146a, IL-6, and TNF-*α*

The levels of miRNA-146a, IL-6, and TNF-*α* in the peripheral blood of the CAS group were higher than those in the normal group (*P* < 0.05) (Table 2). Furthermore, the levels of miRNA-146a, IL-6, and TNF-*α* significantly increased with increasing degrees of CAS stenosis (*P* < 0.05) (Table 3). Additionally, the levels of miRNA-146a, IL-6, and TNF-*α* of the stable plaque group were significantly lower than those of the vulnerable plaque group (*P* < 0.05) (Table 4).

### 3.4. Correlation Analysis between miRNA-146a and CAS Stenosis, IL-6, and TNF-*α*

**Figure 3:**
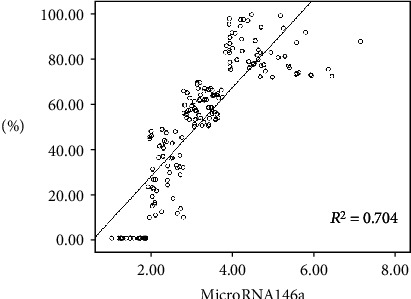
The correlation analysis between microRNA-146a and carotid stenosis.

**Figure 4:**
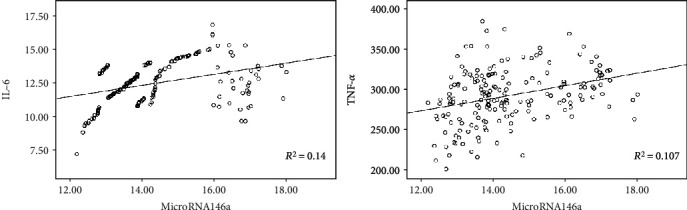
The correlation analysis between microRNA-14a and IL-6 and TNF-*α*.

The results showed a statistically significant positive correlation between miRNA-146a and CAS stenosis (*r* = 0.839, *P* ≤ 0.001) (Figure 3). Additionally, miRNA-146 was significantly positively correlated with IL-6 and TNF-*α* (*r* = 0.374, 0.327, *P* < 0.05) (Figure 4).

### 3.5. miRNA-146a Predicts Vulnerable Plaques

**Figure 5:**
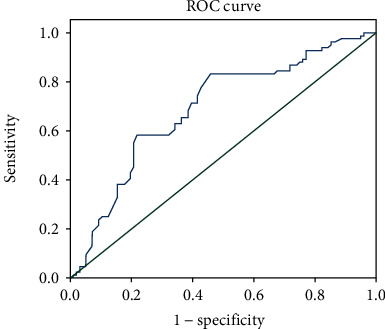
The ROC curve.

To evaluate the predictive value of miRNA-146a for vulnerable plaques in CAS patients, we mapped the ROC curve where miRNA-146a was used as a test variable and plaque vulnerability was assigned as a state variable (1 for vulnerable plaques, 2 for stable plaques). miRNA-146a was found to have a predictive value for vulnerable plaques in CAS patients (AUC = 0.64, 95%CI = 0.613 − 0.769, *σ* = 0.040, *P* < 0.05) (Figure 5).

## 4. Discussion

### 4.1. The Relationship between Inflammatory Cytokines and CAS

The results of this study showed that the levels of IL-6 and TNF-*α* in peripheral blood of patients with CAS stenosis were higher than those in the normal group, which indicates that the inflammatory response plays an important role in the occurrence of CAS. Pathological evidence has shown that CAS exhibits characteristics of inflammation, exudation, hyperplasia, and other inflammatory reactions and that inflammation-related cells are an important part of CAS plaques [9]. In 1999, Ross clearly stated that atherosclerosis is the formation of an intimal inflammatory response [10]. Since then, CAS has gradually become recognized as a chronic inflammatory disease. The inflammatory response not only causes endothelial cell dysfunction and an increase in the production of intercellular adhesion molecules [11] but also promotes the formation of CAS by promoting phagocytosis of macrophages and thus their transition into foam cells. In addition, the inflammatory response can also be passed. Activation of complement-related pathways promotes plaque enlargement [12]. In addition, this study also found that the levels of IL-6 and TNF-*α* gradually increased with increasing CAS stenosis, indicating that the inflammatory response plays an important role in the development of CAS. On the one hand, the inflammatory reaction can promote the rupture of foam cells after phagocytosis of lipids, releasing more lipid content that accelerates the increase in the atherosclerotic core volume; on the other hand, the inflammatory reaction can also cause carotid vascular remodeling, which leads to further narrowing of the vascular lumen. Studies have shown that administration of tranilast in an animal model of vascular stenosis can reduce vascular remodeling and thus improve vascular stenosis [13].

The unstable rupture of plaques in the cerebral vasculature is the main mechanism of cerebral infarction. This study found that the levels of IL-6 and TNF-*α* in peripheral blood of patients with stable plaques were lower than those with vulnerable plaques. Correlation analysis demonstrated a positive correlation between IL-6 and TNF-*α* and plaque vulnerability, which indicates that the inflammatory response also has an impact on the vulnerability of plaques. Studies have shown that vascular smooth muscle cells and macrophages play an important role in plaque vulnerability: vascular smooth muscle cells can maintain the stability of atherosclerotic plaques by producing interstitial collagen, while macrophages can reduce plaque stability by producing a variety of hydrolyzed stroma collagen and extracellular matrix components of matrix metalloproteinase (MMP), such as MMP-9 and MMP-2. The inflammatory response can affect plaque vulnerability through the following mechanisms: the inflammatory response accelerates the oxidation of low-density lipoprotein to ox-LDL, causing more macrophages to phagocytose ox-LDL, which is then transferred to the mononuclear macrophage system. Foam cells change and rupture under the stimulation of the inflammatory reaction, and the released cell components and lipids are deposited locally in the blood vessel. This results in a gradual increase in the lipid core volume, thus a plaque with a larger lipid core volume and more easily broken [14]. Some inflammatory cytokines, such as IFN-*γ*, can inhibit the proliferation and promote apoptosis of smooth muscle cells, which results in decreased extracellular matrix components, thinning of fibrous caps, and thus increased plaque vulnerability [15]. Inflammatory cytokines, such as IL-1 and TNF-*α*, can promote the ability of macrophages to secrete MMP, which results in decreased plaque fiber cap thickness and interstitial collagen composition and thus increased plaque vulnerability [16].

### 4.2. The Relationship between miRNA-146a and CAS

MicroRNA is a class of noncoding single-stranded RNAs of approximately 17−22 nucleotides in length encoded by an endogenous gene [17], which can be identical to the 3- or 5-terminal untranslated region of mRNA of the corresponding target gene. Incomplete base complementary pairing is involved in the development of the disease, as it inhibits the translation or degradation of the corresponding mRNA. In recent years, miRNA-146a has been found to be differentially expressed in inflammatory diseases such as sepsis and COPD [18]. While CAS has been shown to be a chronic inflammatory disease, it is not known whether inflammation-related miRNAs are differentially expressed in CAS and can be used as biomarkers for CAS. The results of this study showed that miRNA-146a levels in the stenosis group were higher than those in the normal group and were positively correlated with the degree of CAS stenosis. The ROC curve also showed that miRNA-146a had a good predictive value for plaque vulnerability in CAS patients. Luo et al. also found that miRNA-146a was involved in the occurrence of atherosclerosis in ApoE^−/−^ mice and was positively correlated with the severity of atherosclerosis [19], similar to the conclusion of this study. However, subject to the limitations of experimental conditions, this study only assessed the correlation and did not explore the specific regulatory mechanisms. Future studies should explore the specific underlying mechanisms of miRNA-146a.

### 4.3. The Relationship between miRNA-146a and IL-6 and TNF-*α*

This study revealed a positive correlation between miRNA-146a and IL-6 and TNF-*α*, suggesting that miRNA-146a may have a regulatory role in the inflammatory response in CAS. The miRNA-146a gene locus is located on the 5q33 chromosome, and its allele is involved in inflammatory signaling pathways such as TNF-*α* and CRP. The TLR4 (Toll-like receptor 4) signaling pathway is a common inflammatory signaling pathway in CAS [20]. When an exogenous inflammatory factor binds to the extracellular domain of TLR4, the signal is transduced into the cell via the TLR4 transcellular structure. Subsequently, TLR4 activates IRAK (IL-1 receptor-associated kinase) via the MyD88-dependent pathway, which further activates TRAF-6 (tumor necrosis factor receptor-associated factor 6). Activation of this signaling pathway may cause nuclear factor-*κ*B (NF-*κ*B) inhibitors to be degraded such that NF-*κ*B is indirectly activated, followed by inflammatory cytokines such as IL-1. With the involvement of NF-*κ*B, TNF-*α* is activated, and miRNA-146a can bind to the 3-terminal noncoding region of IRAK and TRAF-6 via base pair pairing, thereby negatively regulating the above inflammatory cytokines [21]. This indicates that miRNA-146a exerts an inhibitory effect on inflammation and should be inversely correlated with related inflammatory cytokines. This seems to contradict the results of this study, but careful observation found that the correlation coefficient of miRNA-146a and IL-6 and TNF-*α* was not high (*r* = 0.374, 0.327, *P* < 0.01). There are more possible reasons for this. First, the subjects of the study were different; other studies investigated single cells, with singular influencing factors, whereas the research subject of our study was humans, and the human body is affected by many factors. Second, due to the small number of miRNAs and the large number of target genes, the related miRNAs can have various biological functions, and thus, there must be cross-regulation between different miRNAs. In addition to the negative regulation of miRNA-146, the inflammatory cytokines of CAS patients in this study are also positively regulated by other miRNAs. We have not further controlled miRNA-146a or other positive regulatory miRNAs. The correlation analysis in this paper represents the specific effect of positive and negative regulation of miRNA synthesis. This study only performed correlation analysis, and the specific regulatory mechanisms of miRNA-146a on inflammatory cytokines in CAS remain to be further studied.

## Figures and Tables

**Table 1:** Logistic analysis of CAS risk factors.

	*β*	*σ*	Wald	*P*	OR
Hypertension	1.534	0.335	21.027	≤0.001	4.638
LDL-C	1.087	0.402	7.326	0.007	2.965
HDL-C	-4.179	0.660	40.089	≤0.001	0.015

**Table 2:** The comparison of microRNA expression between the CAS group and normal group.

Item	CAS group	Normal group	*t*	*P*
MicroRNA-146a	3.29 ± 1.18	2.48 ± 0.81	2.15	0.032
IL-6 (ng/l)	12.48 ± 1.55	11.41 ± 1.34	4.84	<0.01
TNF-*α* (pg/ml)	292.27 ± 33.58	261.28 ± 36.59	2.45	0.014

**Table 3:** Comparison of microRNA-146a between different CAS groups.

Item	*n*	MicroRNA-146a	IL-6 (ng/l)	TNF-*α* (pg/ml)
Mild group	64	2.79 ± 0.97	11.68 ± 1.42	277.37 ± 35.85
Moderate group	62	3.17 ± 0.93^a^	12.54 ± 1.05^a^	295.34 ± 31.80^a^
Severe stenosis group	54	4.02 ± 1.31b	13.35 ± 1.33^b^	306.39 ± 25.19^b^
*P*		≤0.001	≤0.001	≤0.001

Note: a indicates a statistically significant difference compared with the mild group (*P* < 0.05); b indicates a statistically significant difference between the moderate group and the mild group (*P* < 0.05).

**Table 4:** The comparison of microRNA-146a/IL-6/TNF-*α* expression between the stable group and vulnerable plaque group.

Item	Stable group	Vulnerable plaque group	*t*	*P*
MicroRNA-146a	2.84 ± 0.86	3.31 ± 0.89	3.41	<0.001
IL-6 (ng/l)	11.88 ± 1.35	12.35 ± 0.85	2.43	0.015
TNF-*α* (pg/ml)	281.54 ± 37.97	293.33 ± 32.87	2.11	0.035

## Data Availability

All data, models, and codes generated or used during the study appear in the submitted article.
